# The first dinosaurs from the Early Cretaceous Hami Pterosaur Fauna, China

**DOI:** 10.1038/s41598-021-94273-7

**Published:** 2021-08-12

**Authors:** Xiaolin Wang, Kamila L. N. Bandeira, Rui Qiu, Shunxing Jiang, Xin Cheng, Yingxia Ma, Alexander W. A. Kellner

**Affiliations:** 1 Key Laboratory of Vertebrate Evolution and Human Origins, Institute of Vertebrate Paleontology and Paleoanthropology, Chinese Academy of Sciences, Beijing, 100044 China; 2 CAS Center for Excellence in Life and Paleoenvironment, Beijing, 100044 China; 3 University of Chinese Academy of Sciences, Beijing, 100049 China; 4 Laboratory of Systematics and Taphonomy of Fossil Vertebrates, Department of Geology and Paleontology, Museu Nacional/Universidade Federal do Rio de Janeiro , Rio de Janeiro, 20940-040 Brazil; 5 Beijing Museum of Natural History, Beijing, 100050 China; 6 Laboratório de Paleontologia da URCA, Universidade Regional do Cariri, Rua Carolino Sucupira, s/n, Crato, 63100-000 Brazil; 7 College of Earth Sciences, Jilin University, Changchun, 130061 China; 8 Hami Museum, Hami, 839000 China

**Keywords:** Evolution, Palaeontology

## Abstract

The Early Cretaceous Hami Pterosaur Fauna in Northwest China preserves a large number of specimens of the sexually dimorphic pteranodontoid pterosaur *Hamipterus tianshanensis*, including 3D eggs and embryos. During the last decade, several more fossils have been collected in this area, including three somphospondylan sauropod specimens. The first is *Silutitan sinensis* gen. et sp. nov., which consists of an articulated middle to posterior cervical vertebrae series. The second, *Hamititan xinjiangensis* gen. et sp. nov., consists of an incomplete articulated caudal sequence that could be assigned to lithostrotian titanosaurs based on the strongly procoelous caudal vertebrae with lateral concave surface, as well as marked ventrolateral ridges. The third specimen consists of four sacral vertebral elements, apparently unfused, with exposed camellate internal bone and regarded as somphospondylan. Cladistic analyses based on different datasets recovered *Silutitan sinensis* as an euhelopodid closely related to *Euhelopus* and *Hamititan xinjiangensis* as a titanosaur. Besides the pterosaur *Hamipterus* and one theropod tooth***,*** these dinosaurs are the first vertebrates reported in this region, increasing the diversity of the fauna as well as the information on Chinese sauropods, further supporting a widespread diversification of somphospondylans during the Early Cretaceous of Asia.

## Introduction

In last decades, our knowledge about the Cretaceous somphospondylan sauropods taxa is increasing at high rates, especially in China. Important somphospondylan taxa have been reported from different China provinces, including the Early Cretaceous *Gobitian*, *Qiaowanlong*, *Daxiatitan*, *Yongjinglong*, *Huanghetitan liujiaxiaensis* from Gansu^1–5^, *Euhelopus* from Shandong^6,7^, some isolated titanosauriform teeth and *Dongbeititan dongi* from Liaoning^8,9^, *Liubangosaurus* from Guangxi^10^, *Ruyangosaurus*, *Baotianmansaurus*, *Xianshanosaurus,* and “*Huanghetitan” ruyangensis* from Henan (^11–15^, although *Baotianmansaurus* has been considered Late Cretaceous in age), and *Borealosaurus* from Liaoning^16^; and the Late Cretaceous *Jiangshanosaurus* and *Dongyangosaurus* from Zhejiang^17,18^, *Zhuchengtitan* from Shandong^19^, *Gannansaurus* from Jiangxi^20^, and *Huabeisaurus* from Shanxi^21,22^. Important somphospondylan taxa were also reported in other East and Southeast Asian countries, including Thailand^23^ and Mongolia^24–29^.

One of the most important areas for vertebrate fossils from China was found in the Tugulu Group of the Junggar Basin, north of the Tian Shan Mountains in Xinjiang, northwestern China. Most of the material came from the Lower Cretaceous lacustrine deposits which yielded several vertebrate fossils since the last century^30–32^. The fossils content consists mainly of the pterodactyloid pterosaurs *Dsungaripterus* and *Noripterus*, several dinosaurs, such as the derived stegosaurian *Wuerhosaurus*^31^, the ceratopsian *Psittacosaurus*^33,34^, the alvarezsaurid *Xiyunykus*^35^, the carcharodontosaurid *Kelmayisaurus*^32,36^, and the coelurosaurs *Tugulusaurus* and *Xinjiangovenator*^31,32^. The paleobiota of this area is known as the Wuerho Pterosaur Fauna^31^. The sole sauropod species described from the Tugulu Group so far is *Asiatosaurus*, composed by a tooth, three incomplete cervical vertebrae and multiple ribs, regarded by some as *nomen dubium* (e.g.^37^).

**Figure 1:**
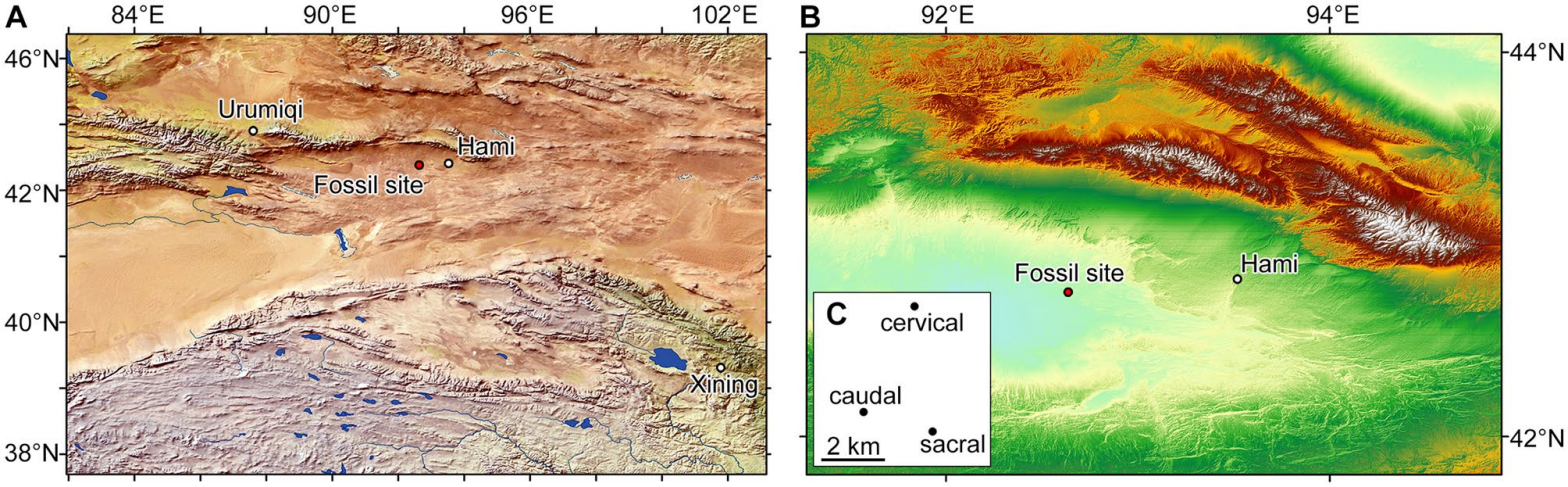
Map showing the fossil site where the new sauropod dinosaur specimens were collected (**A**,**B**), and the relative positions of these three specimens (**C**).

Recently, some interesting fossil sites have been discovered in the Early Cretaceous deposits of the Hami Gobi, more specifically from the Shengjinkou Formation of the Tugulu Group, which is distributed in the Turpan-Hami Basin, south of the Tian Shan Mountains in Xinjiang, China (^38–41^; Fig. 1). It consists mainly of a large number of pterosaurs, including 3D eggs^40^ and embryos^39,41^ of the sexually dimorphic pteranodontoid pterosaur *Hamipterus tianshanensis*. This is one of the few pterosaur bone beds known to date^42^, and provides relevant information on reproduction, development, and habit of these pterosaurs^38–41,43,44^.

Field activities in the Hami region occasionally revealed the presence of other vertebrates, particularly sauropods, that were found by the Hami field team of the IVPP. Among the most important specimens are an incomplete sacral series collected in 2008 (IVPP V27875), a partial tail with an associated theropod tooth recovered in 2013 (HM V22), and a partial articulated sauropod cervical vertebrae series that was associated with a lower jaw of *Hamipterus* in 2016 (IVPP V27874). Here we describe these materials, that represent a new euhelopodid sauropod (IVPP V27874) and a new titanosaur (HM V22). We also include information of the third specimen (IVPP V27875), an indeterminate somphospondylan. All the studies provide new morphological information of Asian somphospondylan sauropods.

### Geological settings

The Tugulu Group is composed of medium-to-fine grained grey-green sandstones alternating with red to brown–red mudstones beds. In Turpan-Hami Basin, this stratigraphic unit includes, from bottom to top, the Sanshilidadun, Shengjinkou, and Lianmuqin formations^38,45^. The Hami Pterosaur Fauna comes from the Shengjinkou Formation whose lacustrine sedimentary sequence is mainly composed by the gray-white sandstones, within some tempestite interlayers made up of brown mudstone breccias^38,40,45^. While almost *Hamipterus* specimens and their eggs were found in tempestite interlayers^40,41^, the new sauropod specimens were discovered in lacustrine sandstones. The three sauropod specimens were collected from different sites which are 2–5 km away from each other, all showing the presence of *Hamipterus*. The horizon where the cervical vertebrae sequence (IVPP V27874) was collected is particularly rich in pterosaur specimens. The layers from which the other two sauropod specimens (HM V22 and IVPP V27875) were recovered are positioned about 2 to 3 m higher than the latter. Occasionally, isolated bones of both, sauropods and theropods (undescribed) are also found.

## Background

Other fossil sites close to the Hami region are the strata from the Junggar Basin, especially the outcrops near the Mazong Mountain^46^. Among the sauropod remains known from the Mazong Mountain (also called the “Mazongshan area”^1^), are two well-known titanosauriform taxa^46^: *Gobititan shenzhouensis* You et al., 2003 (from Gongpoquan Basin) and *Qiaowanlong kangxii* You & Li, 2009 (from Yujingzi Basin). From the Junggar Basin, most sauropod taxa are mamenchisaurids (*Tienshanosaurus chitaiensis* Young, 1937; *Mamenchisaurus sinocanadorum* Russell & Zheng, 1994, and *Klamelisaurus gobiensis*, Zhao, 1993, sensu^47^). *Fushanosaurus qitaiensis* Wang et al*.,* 2019, a putative titanosauriform, was recovered from the Shishugou Formation and is based on a right femur^48^. From all listed somphospondylans recovered from the Mazong Mountain, the only one that shows comparable elements with one of the specimens described here (IVPP V27874—*Silutitan*) is *Qiaowanlong*.

Recently, during the redescription of *Klamelisaurus* by Moore et al.^47^, the phylogenetic analyses conducted using the dataset from Carballido et al.^49^ and González-Riga et al.^50^ found the “core *Mamenchisaurus*-like taxa” (*Klamelisaurus* and *Mamenchisaurus sinocanadorum* included), and some taxa (e.g., *Euhelopus*) thought to represent somphospondylans outside of Neosauropoda^47^, which is inconsistent with most sauropod cladistics analysis literature^49–54^. It is important to note to the authors highlighted a need for further redescriptions due to conflicting phylogenetic results^47^, as well as revisions of these sauropods (particularly *Mamenchisaurus* and *Omeisaurus*). The comparisons with this “core- *Mamenchisaurus*-like taxa” and consequently full revision of the anatomy and systematics of mamenchisaurids is beyond the scope of this paper.

The specimens described here (IVPP V27874, IVPP V27875 and HM V22) are compared with the following somphospondylans: *Abdarainurus barsboldi* Averianov & Lopatin, 2020^55^, *Baotianmansaurus henanensis* Zhang et al*.,* 2009^13^*; Daxiatitan binglingi* You et al*.,* 2008 ^3^; *Dongyangosaurus sinensis* Lü et al., 2008^18^; *Erketu ellisoni* Ksepka & Norell, 2006^28,29^, *Euhelopus zdanskyi* (Wiman, 1929)^7,47^, *Gobititan shenzhouensis* You et al., 2003^1^; *Huabeisaurus allocotus* Pang and Cheng, 2000^22^; *Huanghetitan liujiaxiaensis* You et al. 2006^5^; “*Huanghetitan” ruyangensis* Lü et al., 2007^15^; *Jiangshanosaurus lixianensis* Tang et al*.*, 2001^17,54^; *Phuwiangosaurus sirindhornae* Martin et al. 1994^23,47^ *Qiaowanlong kangxii* You & Li, 2009^2^; *Ruyangosaurus giganteous* Lü et al., 2009^11,12^ and *Yongjinglong datangi* Li et al., 2014^4^.Comparisons are also made with the following titanosaur species: *Andesaurus delgadoi* Calvo and Bonaparte, 1991^56,57^; *Arrudatitan maximus* (Santucci and Arruda-Campos, 2011)^58,59^; *Austroposeidon magnificus* Bandeira et al., 2016^60^; *Bonatitan reigi* Martinelli and Forasiepi, 2004^61^; *Baurutitan britoi* Kellner et al., 2005^62^; *Borealosaurus wimani* You et al. 2004^16^; *Dreadnoughtus schrani* Lacovara et al*.*, 2014^63^; *Diamantinasaurus matildae* Hocknull et al. 2009^64^; *Dongbeititan dongi* Wang et al*.*, 2007^9^; *Epachthosaurus sciuttoi* Powell, 1990^65^; *Gondwanatitan faustoi* Kellner & Azevedo, 1999^66^; *Kaijutitan maui* Filippi et al., 2019^67^; *Lirainosaurus astibiae* Sanz et al., 1999^68,69^ *Malawisaurus dixeyi* Jacobs et al*.*, 1993^70^; *Neuquensaurus australis* (Lydekker, 1893)^71,72^; *Opisthocoelicaudia skarzynskii* Borsuk-Białynicka, 1977^25^; *Patagotitan mayorum* Carballido et al. 2017^49^; *Rapetosaurus krausei* Curry-Rogers and Foster, 2001^73^; *Rinconsaurus caudamirus* Calvo and González-Riga, 2003^74^; *Saltasaurus loricatus* Bonaparte and Powell, 1981^75^; *Tengrisaurus starkovi* Averianov and Skutschas, 2017^76,77^; *Trigonosaurus pricei* Campos et al., 2005^78^, and *Xianshanosaurus shijiagouensis* Lü et al*.* 2009^14^. Additional anatomical comparisons are made with the titanosaurian pelves described by Campos and Kellner^79^ and by Filipini et al*.*^80^, and other eusauropods axial remains^81–83^.

## Results

### Systematic paleontology

SAUROPODA Marsh, 1878

NEOSAUROPODA Bonaparte, 1986

TITANOSAURIFORMES Salgado et al., 1997

SOMPHOSPONDYLI Wilson & Sereno, 1998

EUHELOPODIDAE Romer, 1956 (sensu D’Emic, 2012)

*Silutitan* gen. nov.

ZooBank LSID: urn:lsid:zoobank.org:act:A38DB31D-9375-4D85-A34F-3B099FA19DEF

***Type species.****Silutitan sinensis* sp. nov., type by monotypy.

***Etymology.*** “*Silu*” means the “Silk Road” in Chinese Mandarin pinyin, in memory the great trade routes which connected the East and West. “*titan*” means giant in Greek, symbolic of the large size of this genus.

***Diagnosis.*** The same for the species.

*Silutitan sinensis* new species.

ZooBank LSID: urn:lsid:zoobank.org:act:5486C746-C883-4B9C-BEFB-F711AA122710

***Etymology.*** "*sinensis*" refers to China, in Latin.

**Figure 2:**
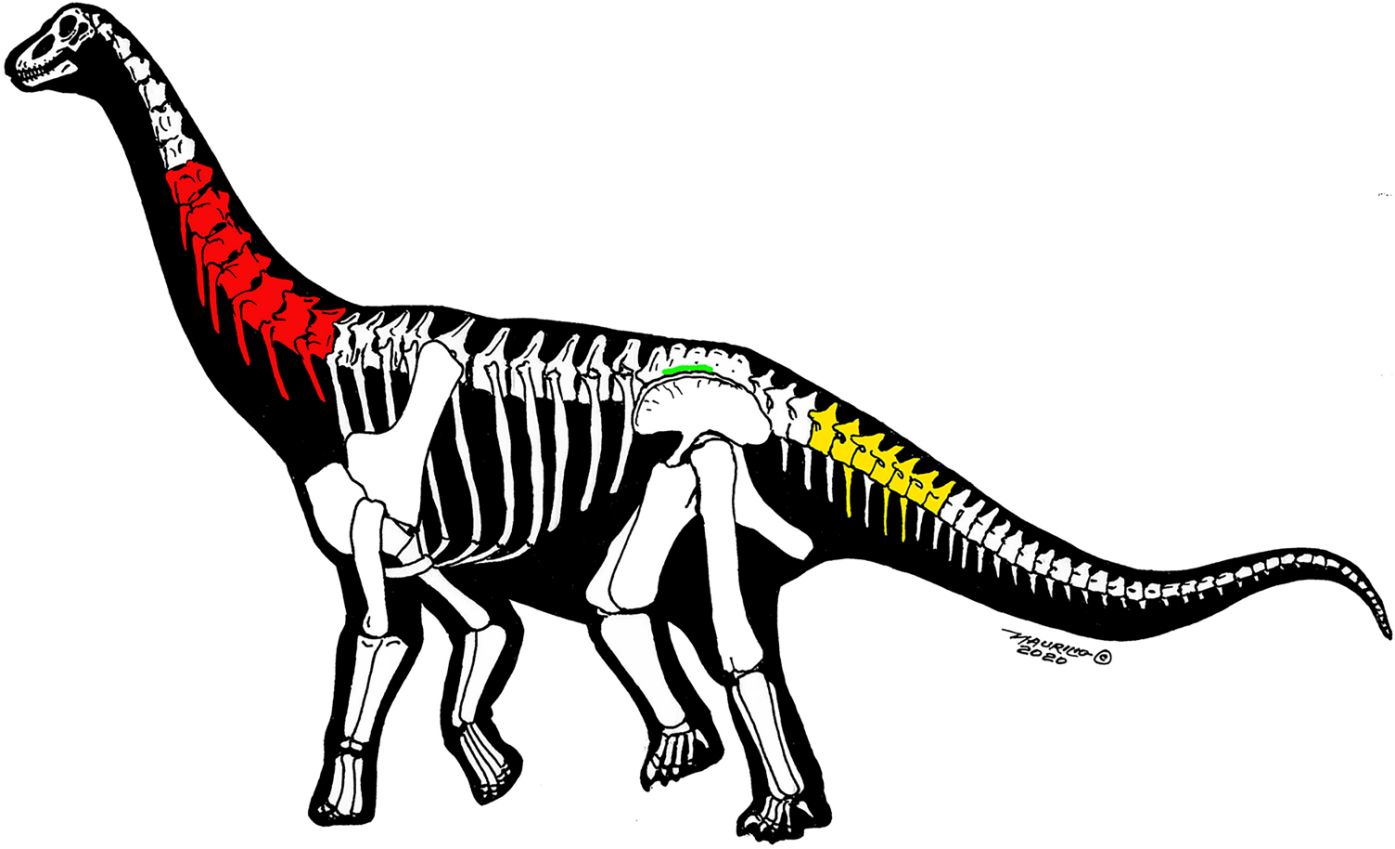
All specimens described in this paper shown in one outline of a generic titanosaur: preserved cervical elements of *Silutitan sinensis* gen. et sp. nov. (IVPP V27874) (red), preserved caudal elements of *Hamititan xinjiangensis* gen. et sp. nov. (HM V22) (yellow) and the preserved sacral elements (IVPP V27875) (green). Image credit: Maurílio Oliveira.

**Figure 3:**
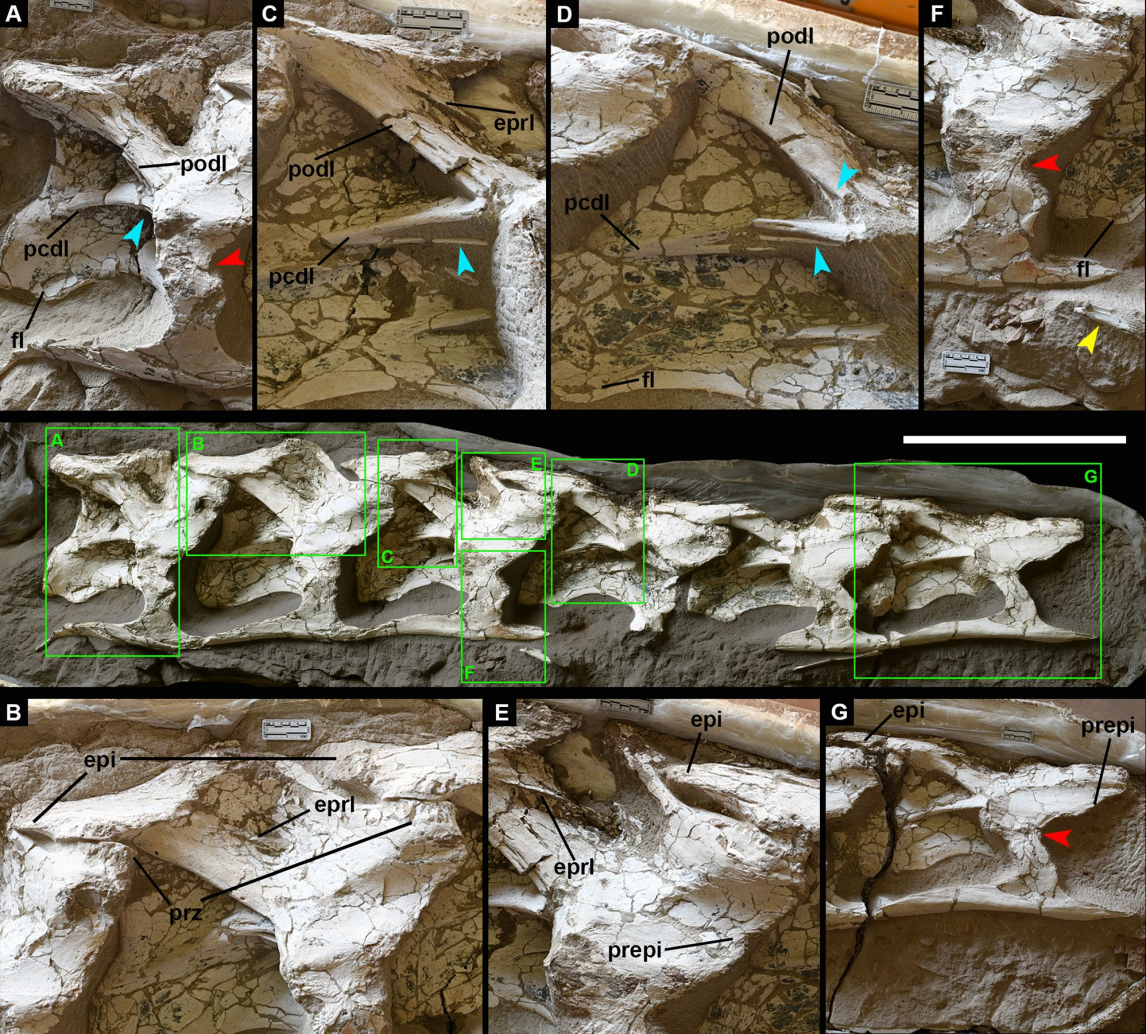
*Silutitan sinensis* gen. et sp. nov. (holotype-IVPP V27874) in left lateral view. Squares separated by letters indicate relevant anatomical details. (**A**) Posteriormost cervical vertebrae, with the bifurcated pcdl (posterior centrodiapophyseal lamina) and podl (postzygodiapophyseal lamina). (**B**) Articulation of cervicals 14 and 15, showing the epiphophysis (epi) and the epipophyseal–prezygapophyseal lamina (eprl). (**C**) Cervical vertebrae 13 showing details of the eprl, podl and pcdl. (**D**) Cervical vertebrae 12 showing details of the podl, pcdl and the fl (flange). (**E**) Cervical vertebrae 12 and 13 with “pre-epipophysis” (prepi), visible eprl and markedly epipopophysis (epi). (**F**) Cervical vertebra 13 showing well developed lateral flange close to the centrum posterior end. (**G**) Cervical vertebra 11, with markedly “pre-epipopophysis” (prepi) together with developed epipophysis. Red arrows show the constricted anteroposteriorly diapophyses-tuberculum contact. Blue arrows highlight laminae bifurcations. Yellow arrow indicates associated pterosaur lower jaw. Scale bar: 50 cm.

***Holotype.*** An articulated series of six cervical vertebrae (IVPP V27874) with almost all cervical ribs, housed at IVPP (Figs. 2, 3; Table 1).

**Table 1:** Selected measurements of *Silutitan sinensis * gen. et sp. nov. (in mm).

	Maximum length	Epipophysis lenght	Maximum height	Neural arch heigth	CPOL height
Cv 10	520	100	260	~ 140	~ 70
Cv 11	490	80	270	~ 150	n.o
Cv 12	510	90	280	~ 190	~ 110
Cv 13	540	550	350	~ 245	~ 140
Cv 14	520	650	410	~ 230	~ 170
Cv 15	455	n.o.	420	280	140

“ ~ ” indicates approximate measurements.

n.o., not observable.

***Locality and Horizon.*** Hami, Xinjiang, China; Lower Cretaceous Shengjinkou Formation (Tugulu Group).

***Diagnosis.*** An euhelopodid sauropod exhibiting the following autapomorphies found in the cervical vertebrae: (1) ventrolaterally bifurcated postzygodiapophyseal laminae [PODL] in middle to posterior cervical vertebrae, (2) anteriorly bifurcated posterior centrodiapophyseal laminae [PCDL] on the four posterior-most cervical vertebrae, (3) parapodiapophyseal laminae [PPDL] forming developed ventral flanges, (4) contact surface of diapophysis and tuberculum in the middle and posterior cervical vertebrae constricted on anterior and posterior faces. It is further characterized by the following combination of characters: cervical vertebrae with developed epipophyses, prezygodiapophyseal laminae anteriorly projected, lateral pneumatic fossae on centra restricted anteriorly, neural arches with two fossae bordered by the epipophyseal-prezygapophyseal laminae, and the neural spines reduced anteroposteriorly.

***Description and comparisons of Silutitan.*** The specimen IVPP V27874 consists of six articulated cervical vertebrae, some with the respective cervical ribs (Fig. 3). All elements are preserved three dimensionally, with the external bone surface complete but, except for the two last, most lack the neural spine. Based on the *Euhelopus zdanskyi* neck, one of the most complete Somphospondyli known to date^7^, *Silutitan sinensis* gen. et sp. nov. (IVPP V27874) represents the cervical sequence 10 to 15, and we will refer as such. The body length is estimated as >20 m by comparison with length of the cervicals of *Euhelopus*^7^.

All elements are strongly opisthocoelous and decrease gradually in length posteriorly, a common condition for sauropods (Table 1). The articular surfaces seem to be mediolaterally wider than dorsoventrally tall (since its still covered by matrix), similar to *Qiaowanlong*^2^ but differing from *Euhelopus*^7^ and *Erketu*^28,29^. As expected for titanosauriform sauropods and some mamenchisaurids^47^, the vertebrae show camellate pneumatic structure. *Silutitan* has the lateral margin of the centra slightly excavated, with the pneumatic fossae (pleurocoel) restricted anteriorly and thus differing from *Erketu* and *Euhelopus* (Fig. 3). The fossae of the new species are further placed ventral to the diapophysis, which is best observed in cervical vertebra 12. The parapophysis presents its dorsal surface excavated and deflected ventrally as in *Qiaowanlong*^2^, *Euhelopus*^7^ and *Erketu*^28^. The ventral surfaces are concave and show some sharp ridges formed by the parapodiapophyseal laminae (PPDL), that extend along the ventrolateral edges of the centrum, as in *Euhelopus*^7^ but less transversely developed in the latter. The PPDL are more ventrally developed than in *Euhelopus*, forming a flange-like structure, which do not reach the posterior articulation of the centrum (Fig. 3). As in many somphospondylans, *Silutitan* presents low and anteroposteriolly short neural arch in almost all preserved cervicals, with exception of the last one. The low and anteroposteriolly short neural arches are observed for example, in the euhelopodids *Euhelopus*^7^, *Qiaowanlong*^2^ and *Erketu*^28,29^; in the somphospondylan *Phuwiangosaurus*^23^ and in the titanosaurs *Arrudatitan*^58^, *Bonatitan*^61^ and *Trigonosaurus*^78^.

The prezygapophyses are relatively large but with thin prezygodiapophyseal laminae (PRDL). The PRDL is anteriorly projected forming a developed flange (“pre-epipophysis”, sensu^7^), which reaches the articular facet of the prezygapophyses. The PRDL forming a developed flange is observed in the somphospondylans *Euhelopus*^7^, *Erketu*^28,29^, *Phuwiangosaurus*^23^, *Huabeisaurus*^22^ and on the eusauropod turiasaurian *Moabosaurus*^81^. The epipophyseal-prezygapophyseal lamina (EPRL) is present in all recovered cervical vertebrae (Fig. 3). The EPRL is also observed in *Klamelisaurus*^47^, in the somphospondylans *Euhelopus*^7,47^, *Qiaowanlong*^2^, *Phuwiangosaurus*^23^ and in the titanosaur *Kaijutitan*^67^. As in the taxa cited, the EPRL divides the spinodiaphophyseal fossa (sdf) into two subfossae, but more similar to the condition observed in *Euhelopus*^7^, two subfossae present, one located dorsally (sdf1) and the other ventrally (sdf2). The EPRL is absent in *Erketu* and only present on the posteriormost cervical vertebrae of *Kaijutitan*^67^ and in *Qiaowanlong*^2^, as in some middle to posterior cervical elements of *Phuwiangosaurus*^23^. Moore et al*.*^47^ presents an extensive comparative anatomy of the EPRL among *Klamelisaurus, Euhelopus*, *Kaijutitan*, and *Phuwiangosaurus*, as well as other sauropod taxa. But in *Kaijutitan* and *Phuwiangosaurus*, the EPRL is almost vertically oriented, while this structure is diagonal in *Qiaowanlong*, and horizontal in *Euhelopus* and *Silutitan*.

The diapophyses are relatively short, directed laterally and curved ventrally, as in several somphospondylans (e.g.^2,7,28,29^). Also, the diapophyses-tuberculum contact surface is constricted anteroposteriorly in the middle and posterior cervical vertebrae. Albeit this feature is presented in 8th cervical of *Erketu* (^29^: Fig. 1C) and in the posterior-most cervical of *Euhelopus* (^7^: Fig. 11) and *Daxiatitan* (^3^: Fig. 1a), we note that is not the same condition as observed in *Silutitan*. In *Euhelopus*, *Daxiatitan* and *Erketu*, the “constriction” is presented solely on anterior face at the contact surface of diapophysis and tuberculum, while in *Silutitan* it is presented on the anterior and posterior faces (Fig. 3).

The new taxon shows a developed posterior centrodiapophyseal lamina (PCDL) that is directed ventroposteriorly and bifurcated in cervical 11 to 15. Except for the lognkosaurian titanosaur from Brazil, *Austroposeidon*, (MCT 1628-R, 52), the bifurcation of the PCDL (Fig. 3) in *Silutitan* is unique. The bifurcation of the PCDL is placed close to the diapophysis and not as posterior as in *Austroposeidon*, where it originates on the centrum and is not as deep^60^. Also, since *Austroposeidon* has this feature observable in the posterior-most preserved cervical vertebra, it is unknown if the bifid PCDL was presented along the four last cervical vertebrae (as in *Silutitan*), what clearly differentiates *Silutitan* from the Brazilian species. Still regarding the PCDL, this structure differs in the new species from *Qiawanlong* where it is more horizontal^2^. The postzygodiapophyseal fossae (PODF) in all vertebrae are not so deep as in other somphospondylians but are well delimited by the inclined postzygodiapophyseal laminae (PODL) and posterior centrodiapophyseal laminae (PCDL). The cervical ribs, in turn, are present (although not complete) in all elements, except in the 12th.

The inclination of the postzygapophyses of the preserved cervical vertebrae shows some differences, with the last preserved element being more ventrolaterally directed, as observed in the posterior cervical vertebrae of other sauropods (e.g.^78^). The articulation surfaces of the postzygapophyses can only be observed in the last preserved cervical element, where they are flattened. The postzygapophyses have a stout centrodiapophyseal lamina (CPOL) as in *Euhelopus* and *Yongjinglong*^4^. The postzygodiapophyseal lamina (PODL) is elongated and thick, being proportionally more elongated in the new taxon compared to *Erketu*, *Euhelopus* and *Qiaowanlong*. *Silutitan* differs from *Erketu* and *Qiaowanlong* by showing a ventrolaterally bifurcated PODL, a feature previously discussed by Moore et al.^47^ in *Euhelopus*, *Klamelisaurus* and several “core *Mamenchisaurus*-like taxa”. However, in *Euhelopus* and *Klamelisaurus* the ventrolaterally bifurcated PODL is observed solely on the posterior-most cervical vertebrae, while in *Silutitan* this feature is observed in the 9th, 10th, 12th, 14th and 15th element (Fig. 3), being less developed in the anterior-most cervical vertebrae. This persistence of the ventrally bifid PODL along most of the cervical vertebrae of *Silutitan* is considered an autapomorphy since, to our knowledge, this feature is not observed in any other taxa.

*Silutitan* also presents a variation of the development of the ventrally bifid PODL along the cervical vertebrae sequence. On the anterior-most vertebrae preserved, the bifurcation of this lamina is restricted more anteriorly and positioned almost exclusively ventrally; but on the posterior-most cervical, the bifurcation extends in length and becomes more ventrolaterally than the other cervical vertebrae. We regard the development of the ventrolaterally bifurcated PODL through the cervical series unique to *Silutitan*.

*Silutitan* presents, as other sauropods, developed epipophyses on the dorsal surface of the postzygapophyses, but unlikelythe euhelopodids *Euhelopus zdanskyi*^7^, *Erketu ellisoni*^28,29^, *Phuwiangosaurus*^23^ and *Qiaowanlonq*^2^, the epipophyses of *Silutitan* are elongated*.* The neural spines are well preserved in cervical 14 and 15, showing that they are low and reduced anteroporsteriorly.

An incomplete lower jaw of a pterosaur was recovered associated with this specimen (Fig. 3F). Despite its incompleteness, this specimen shows the same anatomy of the sole pterosaur collected in this region, *Hamipterus tianshanensis*^38^, and is therefore referred to this taxon.

SOMPHOSPONDYLI Wilson & Sereno, 1998

TITANOSAURIA Bonaparte & Coria, 1993

*Hamititan* gen. nov.

ZooBank LSID: urn:lsid:zoobank.org:act:942E2753-D90A-403C-97B2-6C2EEB4B3A95

***Type species.****Hamititan xinjiangensis* sp. nov., type by monotypy.

***Etymology.****“Hami”* refers to Hami city where the specimen was found, “*titan*”, from the giants of the Greek myths and commonly used to name titanosaur taxa.

***Diagnosis.*** The same for the species.

*Hamititan xinjiangensis* new species.

ZooBank LSID: urn:lsid:zoobank.org:act:FC7C98B2-B846-47C7-94FA-0513E84A9FF2

***Etymology. *** “xinjiangensis”, refers to Xinjiang, China.

**Figure 4:**
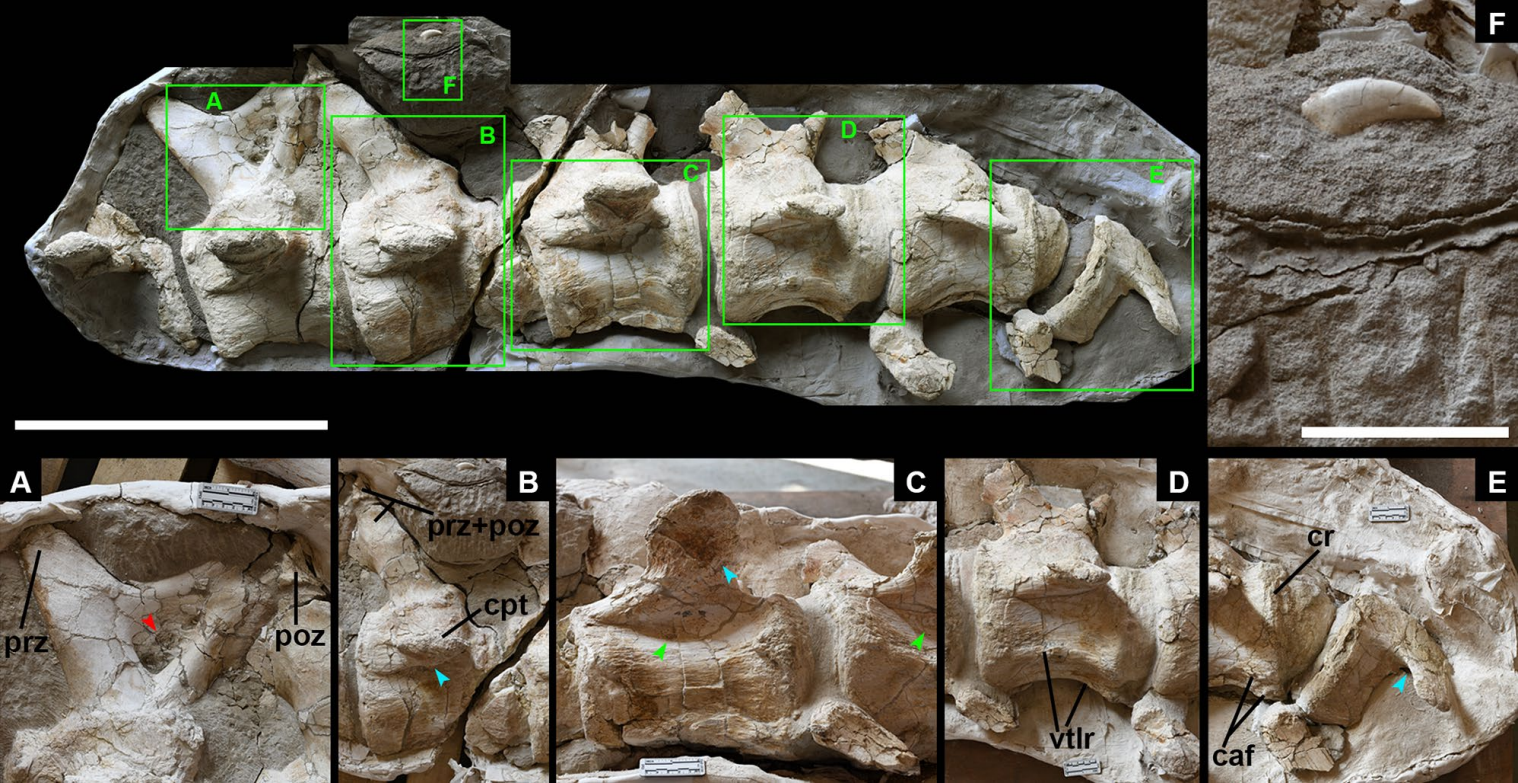
*Hamititan xinjiangensis* gen. et sp. nov., caudal sequence (HM V22) in right lateral view. Squares separated by letters indicate relevant anatomical details. (**A**) Neural arch of the 6th caudal showing pneumatic fossa, open inner small cavities, and the preserved prezygapophysis (prz) and postzygapophysis (poz). (**B**) Detail of the prezygapophyseal (prz) and postzygapophyseal (poz) articulations, and the upward oriented caudal transverse process (cpt). (**C**) Detailed of the caudal transverse process (cpt), with a smooth tuberosity at its ventral face (blue arrow). Green arrows show excavated lateral centra. (**D**) The pronounced ventrolateral ridges (vtrl) found at the anterior to middle caudal vertebrae. (**E**) Transverse processes abruptly changing from upward to downward on the 10th and 11th caudal vertebrae (blue arrow). The 10th caudal vertebra also shows a distinctive condylar rim (cr), and a developed chevron articulation facet (caf). (**F**) The theropod tooth that was found in association with this specimen. Scale bar: 50 cm for the whole specimen and 5 cm in (**F**).

***Holotype.*** An articulated series of seven anterior to middle caudal (HM V22), including the proximal portions of three chevrons, housed at Hami Museum (Figs. 2, 4; Table 2).

**Table 2:** Selected measurements of *Hamititan xinjiangensis* gen. et sp. nov. (in mm).

	Maximum length	Maximum height	Neural arch heigth	Centrum height
Cd 04	n.o.	n.o.	n.o.	~ 170
Cd 05	~ 210	380	190	190
Cd 06	250	360	176	~ 210
Cd 07	260*	330	~ 140	~ 190
Cd 08	290	272	120	210
Cd 09	320	263	~ 90	220
Cd 10	n.o.	~ 170	n.o.	240

“ ~ ” indicates approximate measurements; “*” indicates bones not fully exposed. n.o., not observable.

***Locality and Horizon.*** Hami, Xinjiang, China; Lower Cretaceous Shengjinkou Formation (Tugulu Group).

***Diagnosis.*** A titanosaur sauropod exhibiting the following autapomorphies: (1) tall neural arches with the neural arch higher than the height of the centrum, (2) neural arch on the anteriormost caudal sagittally expanded, (3) deep postzygapophyseal spinodiapophyseal fossa [POSDF] presenting inner open cavities on the anteriormost caudal vertebrae, (4) transverse processes on most anterior caudal vertebrae directed upwards, (5) abruptly change of orientation of the transverse processes from upward (see 3) to downwards. The new species is further characterized by the following combination of characters: prezygapophyses on the caudal vertebrae projecting mainly anterodorsally; and short transverse processes compressed anteroposteriorly and directed laterally.

***Description and comparisons of Hamititan.*** Based on more complete titanosaur caudal sequences (e.g., *Baurutitan*^61^), the seven caudal vertebrae of HM V22 are interpreted as being the fourth to the tenth and are here referred as such (Fig. 4). The body length of this sauropod is estimated as 17 m long by comparison with the length of the caudal of *Rapetosaurus* and *Opisthocoelicaudia*^25,73^.

The caudal vertebrae are strongly procoelous, differing from *Huanghetitan liujiaxiaensis,* “*Huanghetitan” ruyangensis,* *Baotianmansaurus, Dongyangosaurus, Gobititan, Ruyangosaurus,* where they in general are amphiplatyan^1,5,12,13,15,17,18,54^. *Huabeisaurus* and *Abdarainirus* present opisthocoely [incipient on the former^22,55^], differing from *Hamititan.*

It should be noted that strongly procoelous caudal vertebrae are known for *Daxiatitan*^3^, *Dongbeititan*^9^ and *Xianshanosaurus*^14^. *Hamititan* shares with these taxa the lack of pleurocoels and the prezygapophyses positioned close to the proximal margin of the centrum. All four taxa also show the neural spine oriented posterodorsally. *Hamititan* differs from *Daxiatitan*^3^ and *Dongbeititan*^9^ by showing well-marked ventrolateral ridges. Although such ridges are also recorded in *Xianshanosaurus*^54^, the latter differs from *Hamititan* by having longer transverse processes that are also more horizontal^14^. Lastly, *Hamititan* differs from these three taxa by having stouter prezygapophyses^3,9,14^. Furthermore, *Hamititan* shows an abrupt change of the orientantion of the transverse processes throughout the caudal series.

The new taxon lacks internal spongy bony tissue as many titanosaurs^84^. The procoelous caudal vertebrae is not exclusively present in titanosaurs and has been recorded in several eusauropods such as *Mamenchisaurus* and *Wamweracaudia keranjei*^53^, the turiasaurian *Moabosaurus utahensis*^81^. However, in lithostrotian titanosaurs the centra presents at the condylar convex a distinct rim, which separates the condyle from the lateral surface of the main body of the centrum^82^. This feature is also observed in the anterior caudal and middle vertebrae of some derived titanosaurs, such as *Trigonosaurus* and *Baurutitan* and the unnamed titanosaur NHMUK R5333^82^. The new Chinese species has the ventral surface of the centrum slightly concave anteroposteriorly (Fig. 4), as in many lithostrotians, such as *Arrudatitan*^58,59^, *Baurutitan*^62^, *Daxiatitan*^3^, *Dreadnoughtus*^63^, *Dongbeititan*^9^ *Gondwanatitan*^66^, *Rinconsaurus*^74^ NHMUK R5333^82^, and *Xianshanosaurus*^14^.

*Hamititan* also presents the ventrolateral ridge as in the somphospondylans *Abdarainurus*^55^ *Huabeisaurus*^22^ and *Phuwiangosaurus*^23^, as well as in the titanosaurs *Andesaurus*^57^, *Arrudatitan*^58^, *Malawisaurus*^70^, *Opisthocoelicaudia*^25^, *Rapetosaurus*^73^; *Rinconsaurus*^74^; *Saltasaurus*^75^ and *Xianshanosaurus* (based on the scorings of^54^). The 4th, 5th and 6th elements have a smooth excavation on the lateral surface of the centrum that does not form a pneumatopore. The chevron facets are poorly developed, being better developed on 8th and 9th vertebrae.

The neural arches are remarkably high when compared with the total height of the vertebrae (at least on 5th and 6th caudal vertebrae). The neural arches are located on the half of the centrum length on more anterior caudal vertebrae but become closer to the anterior half along the caudal series (Table 2), as in *Abdarainurus*^47^. In the most anterior caudal vertebrae, the neural arch is anteroposteriorly short at its base but broader dorsally at its end, showing a sagittal expanded neural spine, similar to *Lirainosaurus*^68,69^ and *Tengrinsaurus*^76,77^. However, *Hamititan* differs from this Spanish titanosaur as the neural arch that does not reach the anterior border of the centrum as in the former (Fig. 4). In lateral view, the neural arch of the caudal vertebra 5th shows a deep postzygapophyseal spinodiapophyseal fossa [POSDF] delimited by equally thick posterior spinodiapophyseal lamina (SPDL), and the postzygodiapophyseal lamina (PODL), which delimit the are much more robust than the normally presented in other sauropods—a feature so far only observed in *Hamititan xinjiangensis* (Fig. 4).

The prezygapophyses and the postzygapophyses are not preserved except for the 5th and 6th. In lateral view, the zygapophyseal pedicels on the 7th, 8th and 9th are strongly curved and directed upwards (Fig. 4), like the anterior to middle caudal vertebrae of *Opisthocoelicaudia* (plate 4, Fig. 1b^25^). In caudal elements 5 and 6, the prezygapophyses are relatively long and project mainly anterodorsally. In the new species, it is more vertically oriented similar to *Phuwiangosaurus*^23^. The postzygapophysis on caudal vertebra 5 projects posterodorsally, almost reaching the posterior margin of the vertebral centrum, such as in *Neuquensaurus*^71^ and the Russian lithostrotian *Tengrisaurus starkovi* [ZIN PH 7/13 and ZIN PH 14/13^76,77^]. The two centroprezygapophyseal lamina (CPRL) and the centropostzygapophyseal lamina (CPOL) are extremely robust and bounds the neural channel. There is no evidence of diapophyseal laminae on the available caudal elements of *Hamititan xinjiangensis*.

The neural spine is partially preserved only on the 5th, where it is strongly directed posterodorsally as in *Bonatitan* [MACN-PV RN 821^53^], *Tengrisaurus starkovi* [ZIN PH 7/13^76,77^], *Abdarainurus barsboldi*^55^, as well as in the saltasaurines such as *Neuquensaurus* (e.g.^71^).

The transverse processes are placed ventral to the neural arch-centrum contact and have a triangular base, presenting a ridge-like rugosity on the ventral surface. In the 4th element they are short and become longer in the subsequent caudal vertebrae. From caudal vertebra 4th to the 9th, the transverse processes are laterally and with slight upward deflection abruptly changing to a downward deflection on the 10th and 11th. To our knowledge, such an odd and abrupt change of the deflection of transverse processes is reported for the first time in sauropods Three proximal ends of chevrons are preserved and found articulated with the 8th, 9th and 10th anterior caudal vertebrae. The proximal process is laterally compressed and curves gently backwards. Since there is no taphonomic evidence of any deformation, we regard the changes of orientation of the transverse process as an anatomical feature characteristic of this species. It should be noted that the morphology of the laminae and the transverse processes are consistent throughout the caudal series, corroborating with our interpretation.

A small theropod tooth was found associated with this caudal sequence (Fig. 4). It is very curved and has no root. The crown is strongly compressed. The subquadrangular denticles are only preserved on the middle of the distal carina.

SOMPHOSPONDYLI Wilson & Sereno, 1998

**Figure 5:**
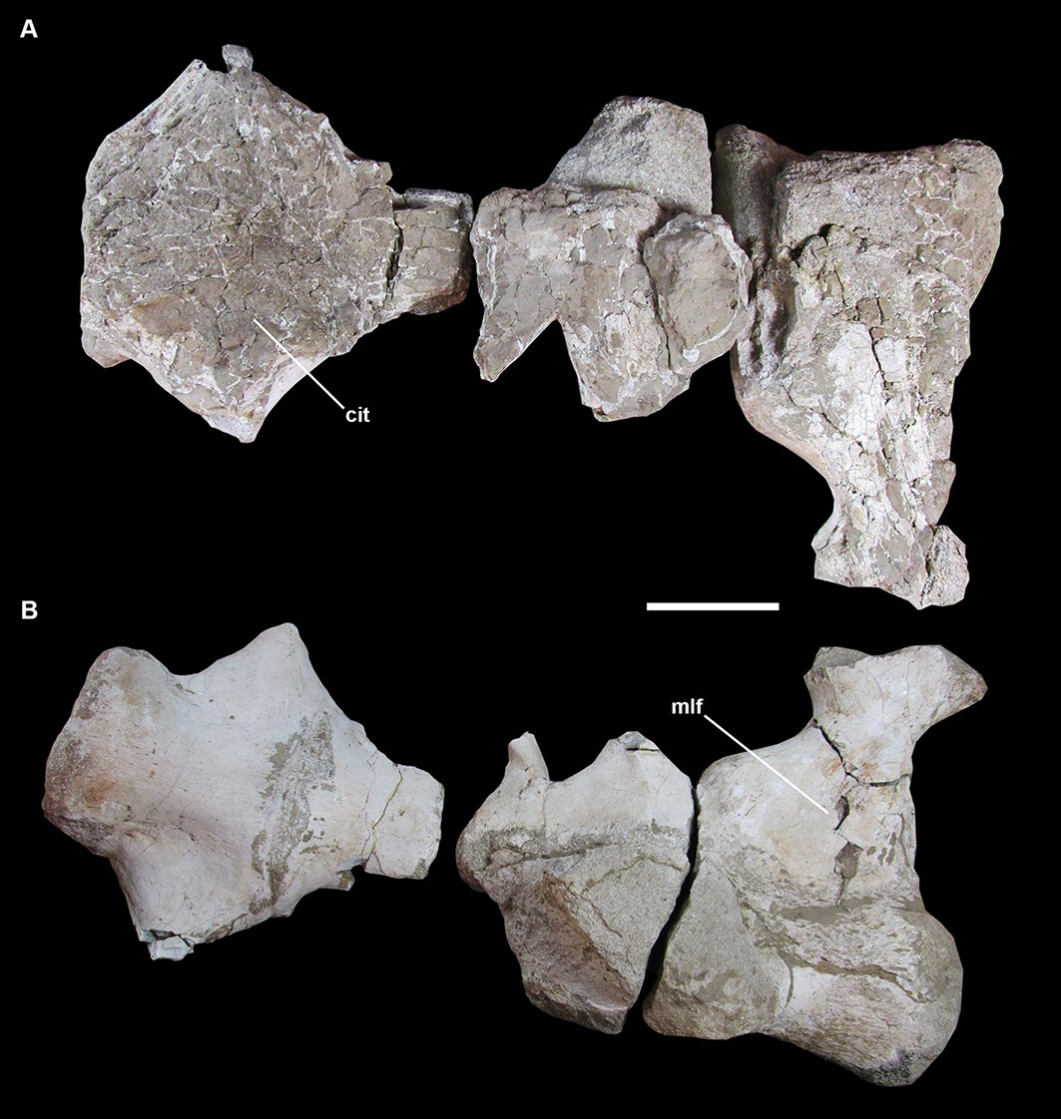
Sacral vertebrae (IVPP V27875), in (**A**) dorsal view, showing the camellate internal tissue (cit) and, (**B**) in ventral view, showing the mediolaterally deep fossa (mlf) on the ventral surface. Scale bar: 10 cm.

***Specimen.*** One incomplete sacrum (IVPP V27875) consisting of 4 fragmentary elements with co-ossified centra and some sacral ribs (Figs. 2, 5).

***Locality and Horizon.*** Hami, Xinjiang, China; Lower Cretaceous Shengjinkou Formation (Tugulu Group).

***Description of IVPP V27875 and comparisons.*** The specimen (IVPP V27875) consists of the remains of at least four incompletes sacral centra with incomplete sacral ribs. They are not completely fused, with clearly marked sutures between sacral 4 and 5. Compared with complete somphospondylan sacra (e.g.^22,64,72,75,78–80^), we regard them tentatively to represent the sacral 2 to 5. The most complete centra are of the sacral 2 and 5, which are short and opisthocoelic (Table 3). In ventral view, the centra are transversely convex (Fig. 5), such as in *Diamantinasaurus*^64^. The dorsal surface is completely eroded with the camellate internal bone exposed. On ventral view, neither centra possess external pneumatic fossae, despite the evidence of closed foramina in sacral 4 and 5 (Fig. 5). The absence of pneumatic fossa distinguishes IVPP V27875 from some somphospondylan like *Phuwiangosaurus*^23^, MLP 46-VIII-21-2^80^, the more derivate titanosaurs *Saltasaurus*^75^ and *Neuquensaurus*^72^. This specimen also differs from *Rapetosaurus*^60^ that shows deep lateral pneumatic foramina.

**Table 3:** Selected measurements of sacral vertebrae (IVPP V27875) (in mm).

	Maximum length	Maximum height	Neural arch heigth	Centrum height
Cs 02	~ 280	n.o.	n.o.	n.o.
Cs 04/05	200	n.o.	n.o.	n.o.

n.o., not observable.

The ventral surface of the centra is concave differing from MLP 46-VIII-21-2^80^ and *Diamantinasaurus*^64^. The sacral ribs are robust, especially in the second sacral vertebra). The best-preserved rib is long, directed laterally and has an extensive and mediolaterally deep fossa on the ventral face (Fig. 5) that is reported for the first time in somphospondylan sauropods. Three well-developed pneumatic foramina are observed on the anteromedial surface of this rib.

### Phylogenetic analysis

**Figure 6:**
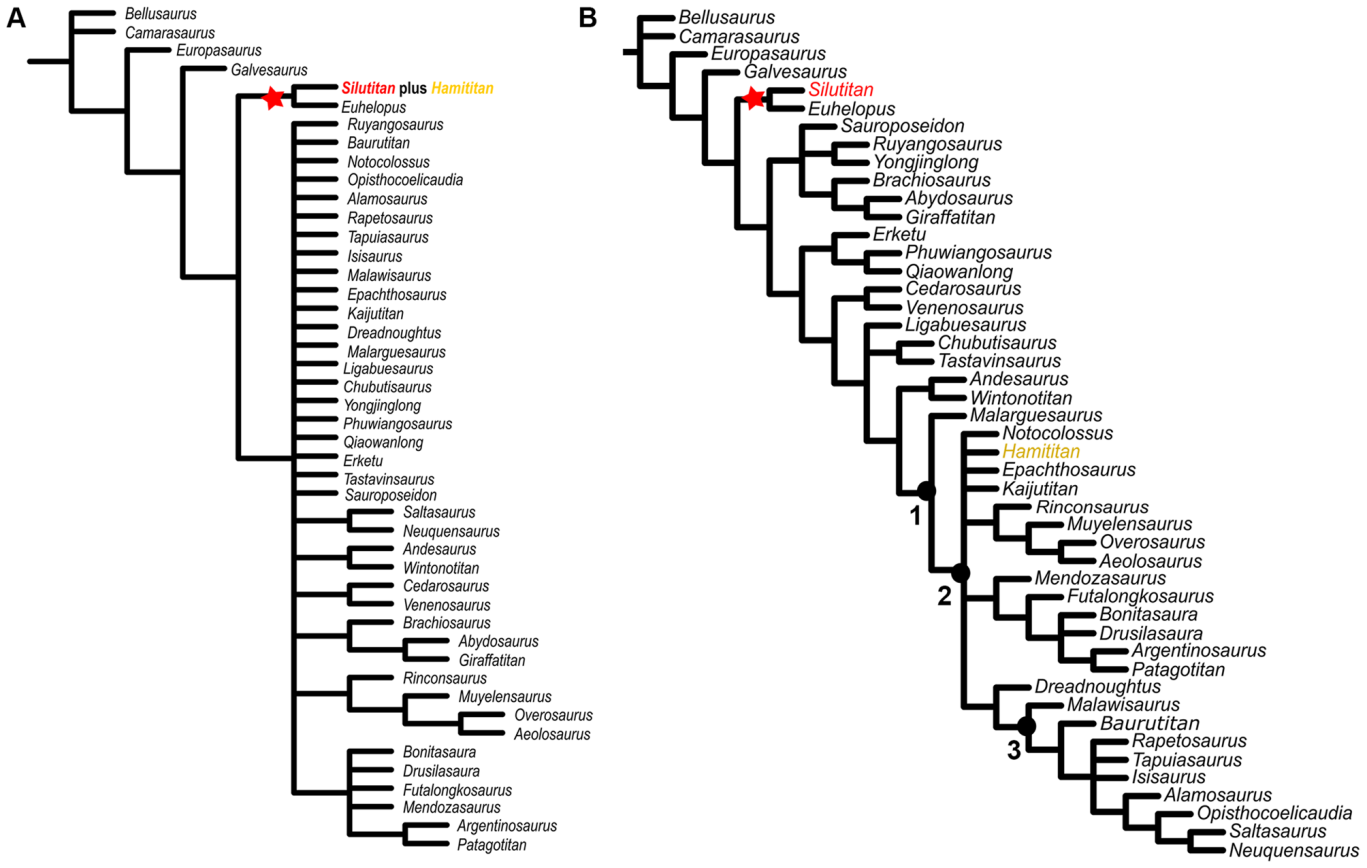
Strict consensus cladogram based on Filippi et al. dataset^67^: (**A**) *Silutitan sinensis* and *Hamititan xinjiangensis* scored as a single taxon; and (**B**) *Silutitan sinensis* and *Hamititan xinjiangensis* scored as two separated taxa. Nodes numbers indicate the clades retrieved: 1. Titanosauria, 2. Colossosauria, and 3. Lithostrotia. *Silutitan sinensis* is highlighted in red and *Hamititan xinjiangensis*, in yellow.

**Figure 7:**
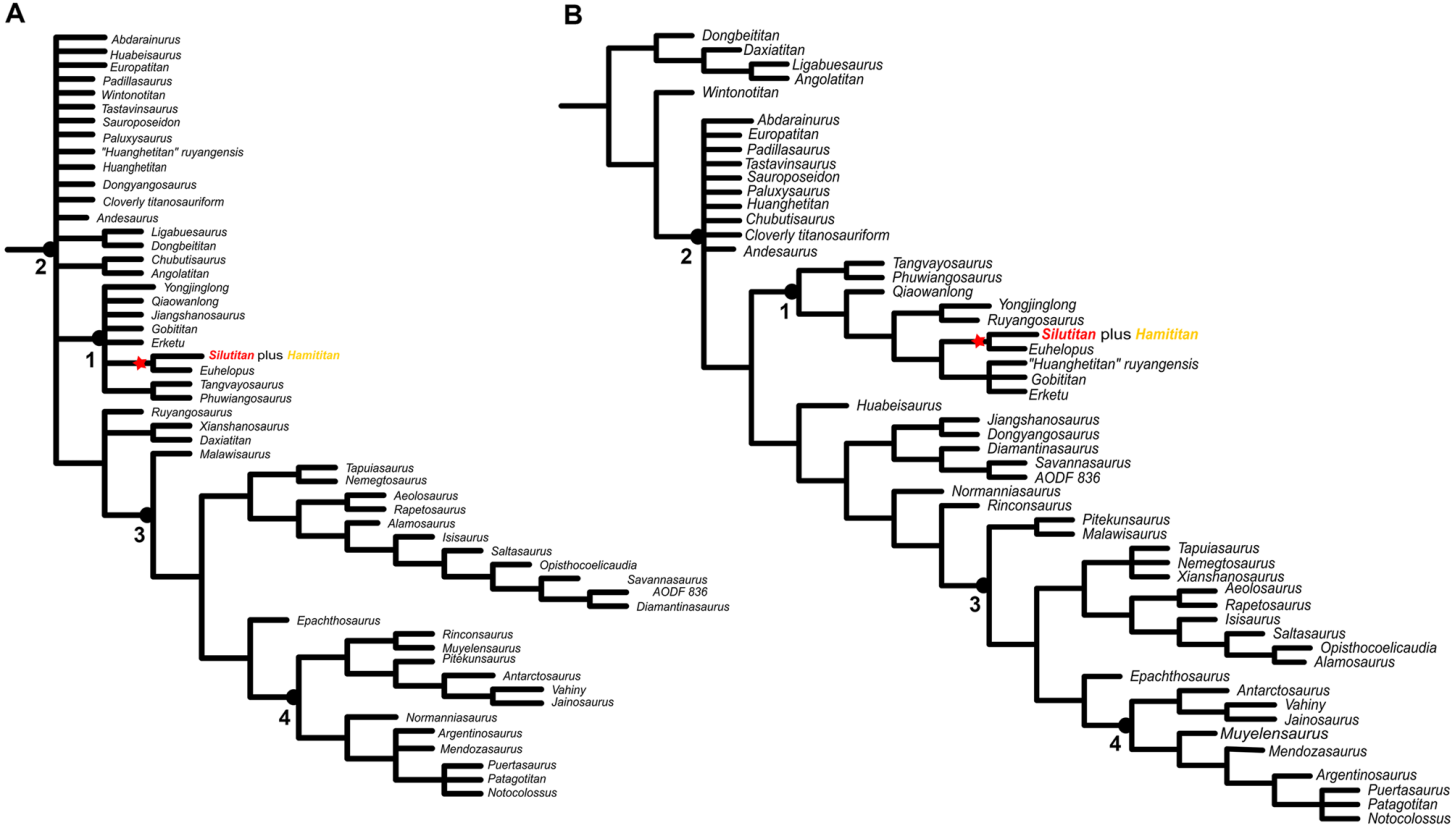
Strict consensus cladogram based on Mannion et al. dataset^54^, with *Silutitan sinensis* and *Hamititan xinjiangensis* scored as a single taxon: (**A**) applying equal weighting; and (**B**) applying extended implied weighting, with k-value 9. Nodes numbers indicate the clades retrieved: 1. Euhelopodidae, 2. Titanosauria, 3. Lithostrotia, and 4. Colossosauria. *Silutitan sinensis* is highlighted in red and *Hamititan xinjiangensis*, in yellow.

**Figure 8:**
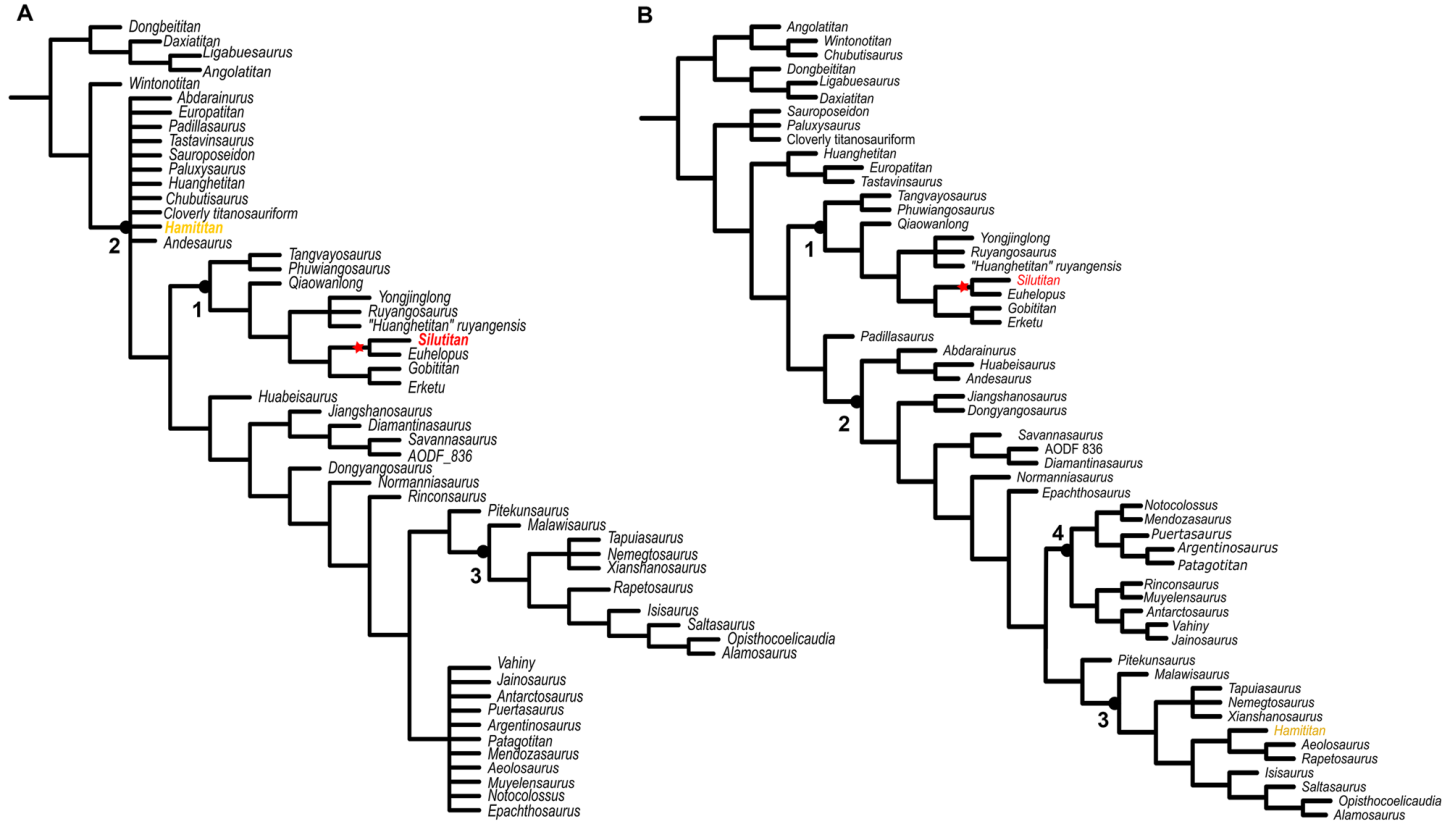
Strict consensus cladogram based on Mannion et al. dataset^54^ with *Silutitan sinensis* and *Hamititan xinjiangensis* scored as two separated taxa: (**A**) applying equal weighting and (**B**) applying extended implied weighting, with k-value 9. Nodes numbers indicate the clades retrieved: 1. Euhelopodidae, 2. Titanosauria, 3. Lithostrotia, and 4. Colossosauria. *Silutitan sinensis* is highlighted in red and *Hamititan xinjiangensis*, in yellow.

The phylogenetic relationships of the somphospondylans *Silutitan sinensis*, *Hamititan xinjiangensis* and IVPP V27875 (Figs. 6, 7, 8) were evaluated using the data matrices focused on Titanosauriformes published by Filippi et al.^67^ and Mannion et al.^53,54^, since those datasets are focused on somphospondylans. While the study of Filippi et al.^67^, focuses on titanosaur interrelationships, Mannion et al.^53,54^ is more concerned with the interrelationships of somphospondylans, especially the basal ones. The data matrix was edited with Mesquite version 3.6^85^ and cladistic analyses were conducted using the software T.N.T. 1.5^86^. Unstable taxa were detected a priori, using the ‘iterpcr’ method in TNT^87^.

Since the materials studied here do not show overlapping elements, we performed three different combinations for each dataset in order to better understand the phylogenetical positioning of each specimen. First, we coded all three specimens as a single taxon. Secondly, we combined *Silutitan* and *Hamititan* as one taxon and excluded the sacral elements. Lastly, we coded *Silutitan* and *Hamititan* as separate taxa and excluded the sacral vertebrae (IVPP V27875) from the analyses.

### Results on Filippi et al. dataset

Considering the matrix of Filippi et al.^67^, we could not include the sacral elements (IVPP V27875) due to lack of scorable characters in this dataset (Supplementary Information). When IVPP V27874 (*Silutitan*) and HM V22 (*Hamititan*) are regarded as representing the same taxon, 29,312 most parsimonious trees (MPTs) with 1359 steps were recovered. The strict consensus tree is much less resolved as in the original study and most of the clades are collapsed. The combined specimens (IVPP V27874 and HM V22) were found as the sister-taxon of *Euhelopus*, sustained only by cervical characters (Fig. 6A).

Scoring *Silutitan* (IVPP V27874) and *Hamititan* (HM V22) as separate taxa resulted in 128 MPTs of 1331 steps. The strict consensus tree is less resolved than the one of the original study. *Silutitan* had no effect on the topology and is recovered as the sister-taxon of *Euhelopus*. This relationship is supported by four characters: the shape and orientation of the parapophysis along the cervical series (122: 0 > 2); the parapophysis shape on middle and posterior cervical vertebrae (147: 0 > 1); the epipophyses shape (129: 0 > 1) and the lateral profile of the neural spine of the posterior cervical vertebrae (149: 0 > 0).

Regarding *Hamititan xinjiangensis* (HM V22), this taxon collapses several clades at the Colossosauria node^52^, generating a polytomy formed by *Kaijutitan*, *Epachthosaurus*, *Hamititan*, and *Notocolossus* (Fig. 6B). If pruning method is applied, one of the pruned taxa is *Hamititan*.

*Hamititan* is nested in Colossosauria supported by the following character states: presence of pneumatized neural arch on anterior caudal vertebrae (221:1) and anterior caudal vertebrae procoelous (230:1).

### Results on Mannion et al. dataset

As in the dataset before, when all three specimens were considered as the same taxon, a large polytomy for Titanosauriformes is recovered (Supplementary Information).

Mannion et al.^54^ used two procedures running their dataset. First, the used equal weighting and after they applied extended implied weighting with different value of k (see^53,54^ for details). Scoring IVPP V27874 (*Silutitan*), HM V22 (*Hamititan*) and IVPP V27875 as a single taxon with equal weighting resulted in 54,450 MPTs with 2672 steps. In this analysis, the composite taxon is also recovered a sister-taxon of *Euhelopus* but retrieved in a large polytomy with other somphospondylans. The inclusion of the two news specimens as a single taxon resulted in a much less resolved consensus tree than the one recovered in the original study^54^. The same results were achieved when eliminating the sacral elements (IVPP V27875) and scoring IVPP V27874 (*Silutitan*), HM V22 (*Hamititan*) as representing the same taxon (Fig. 7A). Running the dataset with the extended implied weighting (k = 9, Fig. 7B), has not changed the relation of *Silutitan* + *Euhelopus* but brought a better resolution within Euhelopodidae, being similar to the topology of the original study by Mannion et al.^53,54^.

Scoring *Silutitan* (IVPP V27874) and *Hamititan* (HM V22) as separate taxa without extended implied weighting resulted in 4148 MPTs with 2616 steps. The strict consensus is less resolved than the one published by Mannion et al.^54^ with only a few clades in Somphospondyli recovered. After pruning the unstable taxa, we find 2615 MPTs and most of the topology of the original study recovered (Fig. 8A). *Silutitan* falls as sister-taxon of *Euhelopus* in the Euhelopodidae, with the remaining taxa of this clade collapsed. *Hamititan* was recovered in a small polytomy with basal titanosaurians, outside Colossosauria (Fig. 8A). The clade formed by *Silutitan* + *Euhelopus* is supported by three characters (Char. 118: 0 → 1; Char. 121: 1 → 0, and Char. 128: 0 → 1). *Hamititan* is nestedad as a basal titanosaurian, what is supported by two synapomorphies (Char. 182: 0 → 1 and Char. 489: 1 → 0).

Running the matrix using extended implied weighting with k = 9 (Fig. 8B) recovered *Hamititan* as a derived titanosaur, as the sister-taxon of (*Aeolosaurus* + *Rapetosaurus*). Comparing the topologies of the consensus trees using both matrices showed the same results regarding *Silutitan sinensis*, always recovered as the sister-taxon of *Euhelopus*, suggesting that they form a separate clade within Euhelopodidae (Fig. 8B).

The position of *Hamititan xinjiangensis*, however, is more instable. Regarding the dataset of Filippi et al.^67^, this taxon is recovered as a Colossossauria, whose position regarding Rincosauria, Lithostrotia and Lognkosauria cannot be established at the time being.

Regarding the dataset of Mannion et al.^54^, *Hamititan xinjiangensis* is recovered in rather extreme positions. When equal weighting is applied, *Hamititan xinjiangensis* is found on the base of Titanosauria while if implied weighing is applied (k = 9), this taxon moves to the top, as the sister-taxon of (*Aeolosaurus* + *Rapetosaurus*). Nonetheless, both datasets, shows that *Hamititan xinjiangensis* is well nested within Titanosauria, distant from *Silutitan sinensis*, corroborating that both represent quite distinct taxa.

## Discussion

### Comments about other East Asian sauropods

The diversity of somphospondylan sauropod genera from the Cretaceous of East Asia increased vastly in the last decades^47,52–54^. Several taxa, however, a lack comparable elements with *Silutitan* and *Hamititan*: *Zhuchengtitan* is represented by a single humerus^19^, *Liubangosaurus* consists of a set of dorsal vertebrae^10^, *Borealosaurus* known by two distal caudals, a humerus and one tooth^16^, and *Gannansaurus* erected based on one middle caudal and one posterior dorsal vertebra^20^.

Regarding other somphospondylans, several East Asian sauropods taxa are classified in the Euhelopodidae, whose interrelationships is still a matter of debate^47,49–54,82^. *Qiaowanlong*, *Gobitian* and *Erketu* are traditionally assigned in this group^7,28,29,53,54,82^ and recent phylogenetic studies have included other taxa such as *Yongjinglong, Liubangosaurus*, *Ruyangosaurus, Huabeisaurus* and “*Huanghetitan” ruyangensis*^22,50,52–54^.

*Silutitan sinensis* can be assigned to the Euhelopodidae based on the presence of a thick EPRL dividing the spinodiaphophyseal fossa into two subfossae, and the pendant cervical ribs. This taxon cannot be compared with the euhelopodids *Gobititan*, *Liubangosaurus*, and “*Huanghetitan” ruyangensis*^1,10,15^ that do not show comparable elements.

*Silutitan* differs from *Qiaowanlong*^2^, that shows the EPRL diagonally oriented, the neural spine bifid, and two fossae on the lateral surface of the centrum of the cervical vertebrae.

The cervical vertebrae of *Yongjinglong* differ from *Silutitan* by having large pleurocoels that almost occupy the entire lateral side of the cervical vertebrae^4^.

*Silutitan* can be distinguished from *Euhelopus*, the first discovered euhelopodid^6,7^ by the absence of a median turbercle on the cervical vertebrate and by having the PODL elongated and ventrolaterally bifurcated in all preserved cervical vertebrae.

*Huabeisaurus* differs from *Silutitan* by the lack of bifurcated PRDL and vertically oriented EPRL, present in the new species.

The cervical vertebrae of *Ruyangosaurus* are not well preserved^11,12^. As far as comparisons are possible, this taxon differs from *Silutitan* by lacking pleurocoels in the anteriormost cervical vertebrae (but having a deep and large pleurocoel in the posteriomost cervical element) and having longer centra. The two posterior-most cervical vertebrae of *Ruyangosaurus* are stouter than the *Silutitan* and show a thick and not bifurcated posterior centrodiapophyseal lamina (PCDL).

*Hamititan xinjiangensis* was recovered as a member of the Titanosauria. Several Early Cretaceous Chinese sauropods had been originally classified in this clade (e.g.^4,17^), but recent studies regarded some as representing other lineages of Titanosauriformes, such as *Gannansaurus*^20^, *Borealosaurus*^16^, *Yongjinglong*^53,54^, *Dongyangosaurus* and *Jiangshanosaurus*^54^. In any case, the caudal vertebrae present in *Gannansaurus*^20^ are from the posterior region of the tail, being amphycoelous and therefore differing from *Hamititan*. The same difference can be observed in the anterior and posterior caudal elements of *Jiangshanosaurus*^54^. *Borealosaurus* also has only posterior caudals^16^, but opisthocoelous. *Huabeisaurus* shows a fairly complete tail^22^, with the anterior being opisthocoelic while the middle and posterior are amphycoelic.

Other titanosaurian taxa recovered from China also differ from *Hamititan*. The putative titanosaur *Dongyangosaurus* has two anterior caudal vertebrae, that shows the anterior and posterior surfaces of the two caudal centra gently concave^18,54^ and proportionally shorter than the ones of *Hamititan*.

*Daxiatitan*^3^, *Xianshanosaurus*^14^ and *Dongbeititan*^9^ show strongly procoelous anterior caudal elements and share with *Hamititan* the lateral surface of the centra lacking pleurocoels, prezygapophyses positioned beyond the proximal margin of the centrum, and the neural spine oriented posterodorsally. *Hamititan* presents well-marked ventrolateral ridges that are absent in *Dongbeititan* and *Daxiatitan*^3,9^. *Xianshanosaurus* further differs from *Hamititan* by having longer and horizontally placed transverse processes^14^, as well as the presence of lateral openings.

Although our phylogenetic analyses did not recover *Hamititan* as an euhelopodid, we have also compared this species with members of this clade. It should be noted that most taxa referred to the Euhelopodidae lack caudal elements, including *Euhelopus*^7^. Depending on the dataset, some authors do regard *Tangvayosaurus, Phuwiangosaurus, Ruyangosaurus* and *Gobititan*, originally described as titanosaurs^1,11,12,23^, as euhelopodids^53,54,82^. They differ from *Hamititan* by having proportionally shorter caudal vertebrae with nearly flat articular ends, presenting an amphiplatyan condition. *Gobititan* shows variation in some of the posterior-most caudals^1^, that can be slightly procoelous, but the anterior elements, as pointed out, differ from *Hamititan*. *Hamititan* further differs from all the above-mentioned taxa by having stouter prezygapophyses, taller neural arches, and presents the unique morphology of the transverse processes that show an abrupt change from being directed upward in the anterior elements to being directed downward in the posterior ones.

### Comments about Euhelopodidae

The Euhelopodidae is a rather problematic clade of sauropod dinosaurs. This name was first proposed by Romer^88^ containing five genera: *Mamenchisaurus*, *Chiayusaurus*, *Omeisaurus*, *Tienshanosaurus*, and *Euhelopus*. The original taxa of Romer have not been recovered as a clade by most of the recent phylogenetic analysis, with *Euhelopus* mostly recovered as Somphospondyli (e.g.^49–54^). The first phylogenetic definition of Euhelopodidae was formulated by D'Emic^89^ as the clade containing “neosauropods more closely related to *Euhelopus zdanskyi* than to *Neuquensaurus australis*” (^89^: pg. 626). Some authors recovered Euhelopodidae as paraphyletic with *Euhelopus* nested far from other euhelopodids, such as *Erketu* and *Qiaowanlong*^49,63^. A more extreme result was obtained by Moore et al.^47^ that recovered *Euhelopus* outside of Macronaria, highlighting the necessity of reviewing this and closely related taxa. In any case, in the phylogenetic analyses presented here, we did consistently recover we recovered *Silutitan sinensis* consistently as the sister-taxon of *Euhelopus*.

### Other associated taxa

Two of the new specimens described here were found associated with elements of other taxa. Close to the 10th cervical vertebrae of *Silutitan sinensis* (IVPP V27874), an incomplete lower jaw attributed to the pterosaur *Hamipterus tianshanensis* is preserved. The association of pterosaur with sauropods have not commonly been reported in the literature (e.g.^90^). It is not clear, however, if there were any more specific palaeoecological interactions between these taxa and this association is likely due to taphonomy.

Regarding *Hamititan xinjiangensis*, a small theropod tooth was observed above the neural arch of the 6th caudal vertebrae. It is the first report of theropod dinosaur discovered in this area. Theropod teeth are commonly found associated with the sauropod remains, generally suggesting that theropods could have fed on their carcasses (e.g.^91,92^). Although this might also have been possible here, no evidence of tooth marks has been observed in this specimen, or on the other sauropod material described here.

## Conclusions

The discovery of *Silutitan sinensis* and *Hamititan xinjiangensis* increased the sauropod diversity of Asia, particularly from an area where these vertebrates are not common. *Silutitan sinensis* is closely related to *Euhelopus*. The existence of a more inclusive clade of similar sauropods (Euhelopodidae) is still a matter of debate and pends on more detailed description of some putative euhelopodid.

*Hamititan xinjiangensis* is one of the few titanosaurian sauropod recovered from Asia, which shows an unusual combination of sauropod features. The presence of two somphospondylan species in the Tugulu Group novel information on somphospondylan evolution and provides further support for a widespread diversification of these sauropods during the Early Cretaceous of Asia.

## Materials and methods

### Anatomical terminology

We used the traditional “Romerian” terminology as proposed by Wilson^93,94^, using for example “anterior” rather than “cranial”, as directional terms. For the identification and designation of vertebral laminae and fossae for Sauropoda we follow the landmark-based scheme proposed by Wilson^93,94^ and Wilson et al.^95^ respectively.

### Heuristic tree search

The datasets of Filippi et al.^67^ and Mannion et al.^53,54^ were analyzed using the “New Technology Search”. The algorithms (“Sectorial Search”, “Ratchet”, “Drift” and “Tree Fusing”) are applied together with the traditional search procedures, such as Wagner Trees, Tree Branch Reconnection (TBR) and Subtree-Pruning-Regrafting (SPR) algorithms, to find the Minimum Length Trees (MLTs). A final round of TBR branch swapping was applied to the best trees obtained at the end of the replicates to find all of the Most Parsimonious Trees (MPTs).

#### Filippi et al. 2019

The matrix consists of 405 characters and 83 taxa, including *Silutitan* and *Hamititan.* The sacral vertebrae (IVPP V27875) were not included since it cannot be scored in this dataset. Characters (14, 61, 100, 102, 109, 115, 127,135,136, 168, 181, 197, 258, 261, 278, 279, 280, 281, 301, 305, 348, 354 and 356) were ordered, as in the original analysis, and using equal weighting of characters.

#### Mannion et al. 2019

The matrix consists of 548 characters and 120 taxa, including the new taxa described here and plus adding the scorings of *Abdarainurus* disponibles in^55^. The characters (11, 14, 15, 27, 40, 51, 104, 122, 147, 148, 195, 205, 259, 297, 426, 435, 472 and 510) were ordered, as in the original analysis and first we use equal weighting of characters. Six unstable taxa were removed after the method of^87^ are IVPP V27875, *Katepensaurus*, *Abydosaurus*, *Losillasaurus, Futalognkosaurus* and *Baotianmansaurus.* As in Mannion et al.^53,54^ additional analyses were performed, using the same pruned matrix and protocol, but applying extended implied weighting in TNT with concavity (k) value of 9.

## Supplementary Information﻿

Supplementary Information 1.

Supplementary Information 2.

Supplementary Information 3.
