# Supplementary material for: The first dinosaurs from the Early Cretaceous Hami Pterosaur Fauna, China
Source: Sci Rep. 2021 Aug 12;11:14962. doi: 10.1038/s41598-021-94273-7 (PMC8361124; doi:10.1038/s41598-021-94273-7)
Supplement: Supplementary file 1 — Supplementary Information 1. [file 41598_2021_94273_MOESM1_ESM.docx]

**Supplementary information**

Wang et al Suppl 1 - analyses protocols.

Wang et al Suppl 2 - TNT file of the dataset of Filippi et al. (2019), with *Silutitan sinensis* gen. et sp. nov. and *Hamititan xinjiangensis* gen. et sp. nov. run as separate taxa.

Wang et al Suppl 3 - TNT file of the dataset of Mannion et al. 2019, with *Silutitan sinensis* gen. et sp. nov., *Hamititan xinjiangensis* gen. et sp. nov. and IVPP V27875 run as separate taxa.

Wang et al Suppl 4 - TNT file of the dataset of Mannion et al. 2019, with *Silutitan sinensis* gen. et sp. nov., *Hamititan xinjiangensis* gen. et sp. nov. and IVPP V27875 run as the same taxon.
