# Supplementary material for: The first dinosaurs from the Early Cretaceous Hami Pterosaur Fauna, China
Source: Sci Rep. 2021 Aug 12;11:14962. doi: 10.1038/s41598-021-94273-7 (PMC8361124; doi:10.1038/s41598-021-94273-7)

**SUPPLEMENTARY MATERIAL FOR**

**The First Dinosaurs from the Early Cretaceous Hami Pterosaur Fauna, China**

Xiaolin Wang^1,2,3^*, Kamila L. N. Bandeira^4^, Rui Qiu^1,3,5^, Shunxing Jiang^1,2^, Xin Cheng^6,7^, Yingxia Ma^8^, Alexander W. A. Kellner^4*^

^1^ Key Laboratory of Vertebrate Evolution and Human Origins, Institute of Vertebrate Paleontology and Paleoanthropology, Chinese Academy of Sciences, Beijing, 100044, China

^2^ CAS Center for Excellence in Life and Paleoenvironment, Beijing, 100044, China

^3^ University of Chinese Academy of Sciences, Beijing, 100049, China

^4^ Laboratory of Systematics and Taphonomy of Fossil Vertebrates, Department of Geology and Paleontology, Museu Nacional/Universidade Federal do Rio de Janeiro, Rio de Janeiro, 20940-040, Brazil

^5^ Beijing Museum of Natural History, Beijing, 100050, China

^6^ Laboratório de Paleontologia da URCA, Universidade Regional do Cariri, Rua Carolino Sucupira, s/n, Crato, 63100-000, Brazil

^7^ College of Earth Sciences, Jilin University, Changchun, 130061, China.

^7^ Hami Museum, Hami, 839000, China

*Corresponding author. Email: wangxiaolin@ivpp.ac.cn (X.W.) and [kellner@mn.ufrj.br](mailto:kellner@mn.ufrj.br) (A.W. K)

**SCORINGS OF THE TAXA ANALIZED HERE**

**1) On Filippi et al. 2019 dataset.**

*Silutitan sinensis* gen. et sp. nov.

?????????????????????????????????????????????????????????????????????????????????????????????????????????????????????????21??311?0??012????1???0011001?00???????????????????????????????????????????????????????01??????????????????????????????????????????????????????????????????????????????????????????????????????????????????????????????????????????????????????????????????????????????????????????????????

*Hamititan xinjiangensis* gen. et sp. nov.

????????????????????????????????????????????????????????????????????????????????????????????????????????????????????????????????????????????????????????????????????????????????????????????????????????????????????????????01?????01130?0?0?0000?00??????0?10?????2?????????????????????????????????????????????????????????????????????????????????????????????????????????????????????????????????????????????????

**2) On Mannion et al. 2019 dataset**

*Silutitan sinensis* gen. et sp. nov.

?????????????????01???????????????????????????????????????????????????????????????????????????????????????????????2?0?1?0001011110110000?110???????????????????????????????????????????????????????????????????????????????????????????????????????????????????????????????????????????????????????????????????????????????????????10001100???????????????????????????????????????????????????????????????????????00001?????????00??????????????????????????????????????0??000000???????????????????????????????????????????????????????????????????????????????????

*Hamititan xinjiangensis* gen. et sp. nov.

?????????????????????????????????????????????????????????????????????????????????????????????????????????????????????????????????????????????????????????????????????????????

??010-011??????00???0010??????010?00???????????????????????????????????????????????????

???????????????????????????????????????????????????????????????????????????????????????????00?000???????????????????????????????????????????????????0?0???????0?????????????????????????????????????????????????????????????????????????0???0???00000?1???????????????????????????????????????0?

Sacral vertebrae (IVPP V27875)

???????????????????????????????????????????????????????????????????????????????????????????????????????????????????????????????????????????????????????????????????????????10???????????????????????????????????????????????????????????????????????????????????????????????????????????????????????????????????????????????????????????????????????????????????????????????????????????????????????????????????????????????????????????????????????????????????????????????????????????????????????????????????????????????????????????????????????????????????????

**RESULTING CONSENSUS TREES**

**1) From Filippi et al. 2019 dataset.**

A) Resulting tree when *Silutitan sinensis* gen. et sp. nov. and *Hamititan xinjiangensis* gen. et sp. nov. are run separately.

B) Resulting strict consensus tree when *Silutitan sinensis* gen. et sp. nov. and *Hamititan xinjiangensis* gen. et sp. nov. are run as a single taxon (scored here as “CombTax”).


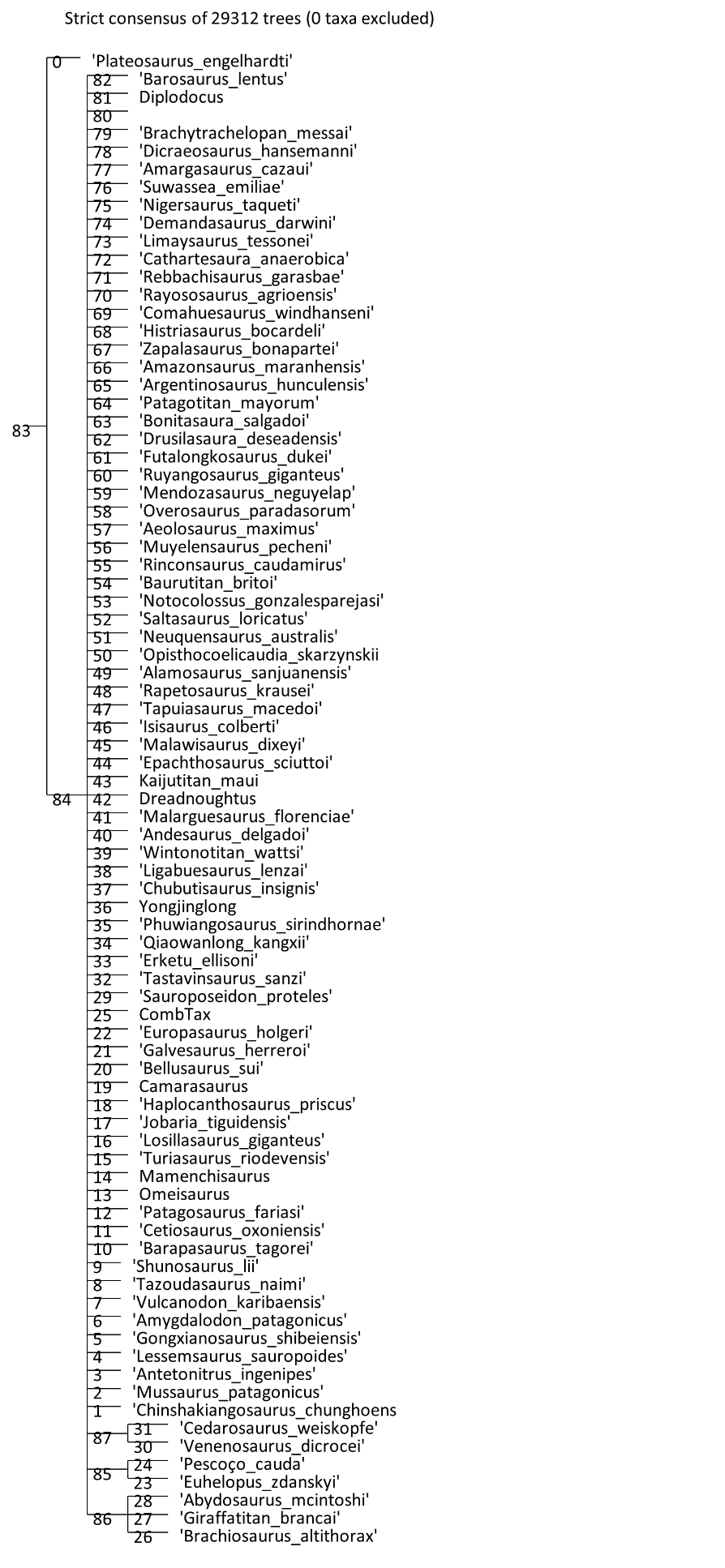


**2) From Mannion et al. 2019 dataset.**

A) Resulting strict consensus tree when *Silutitan sinensis* gen. et sp. nov. and *Hamititan xinjiangensis* gen. et sp. nov. and IVPP V27875 run separated, and no implied weighting was applied (scored here as “Cervical Sequence”, “Caudal” and “Sacral”, respectively).

B) Resulting strict consensus tree when *Silutitan sinensis* gen. et sp. nov. and *Hamititan* *xinjiangensis* gen. et sp. nov. and IVPP V27875 run separated (scored here as “Cervical Sequence”, “Caudal” and “Sacral”, respectively), with implied weighting applied (K= 3).

C) Resulting strict consensus tree when *Silutitan sinensis* gen. et sp. nov. and *Hamititan* *xinjiangensis* gen. et sp. nov. and IVPP V27875 run separated (scored here as “Cervical Sequence”, “Caudal” and “Sacral”, respectively), with implied weighting applied (K= 9)

D) Resulting strict consensus tree when *Silutitan sinensis* gen. et sp. nov. and *Hamititan* *xinjiangensis* gen. et sp. nov. (scored here as “Cervical Sequence” and “Caudal”, respectively), the sacral specimen (IVPP V27875) was excluded and without implied weighting.

E) Resulting strict consensus tree when *Silutitan* *sinensis* gen. et sp. nov. and *Hamititan* *xinjiangensis* gen. et sp. nov. (scored here as “Cervical Sequence” and “Caudal”, respectively), the sacral specimen (IVPP V27875) was excluded, and with implied weighting (K= 9).

F) Resulting strict consensus tree when *Silutitan* *sinensis* gen. et sp. nov. and *Hamititan* *xinjiangensis* gen. et sp. nov. and the the sacral specimen (IVPP V27875), all three specimens scored together (scored here as “CombTax”), as a single specimen.


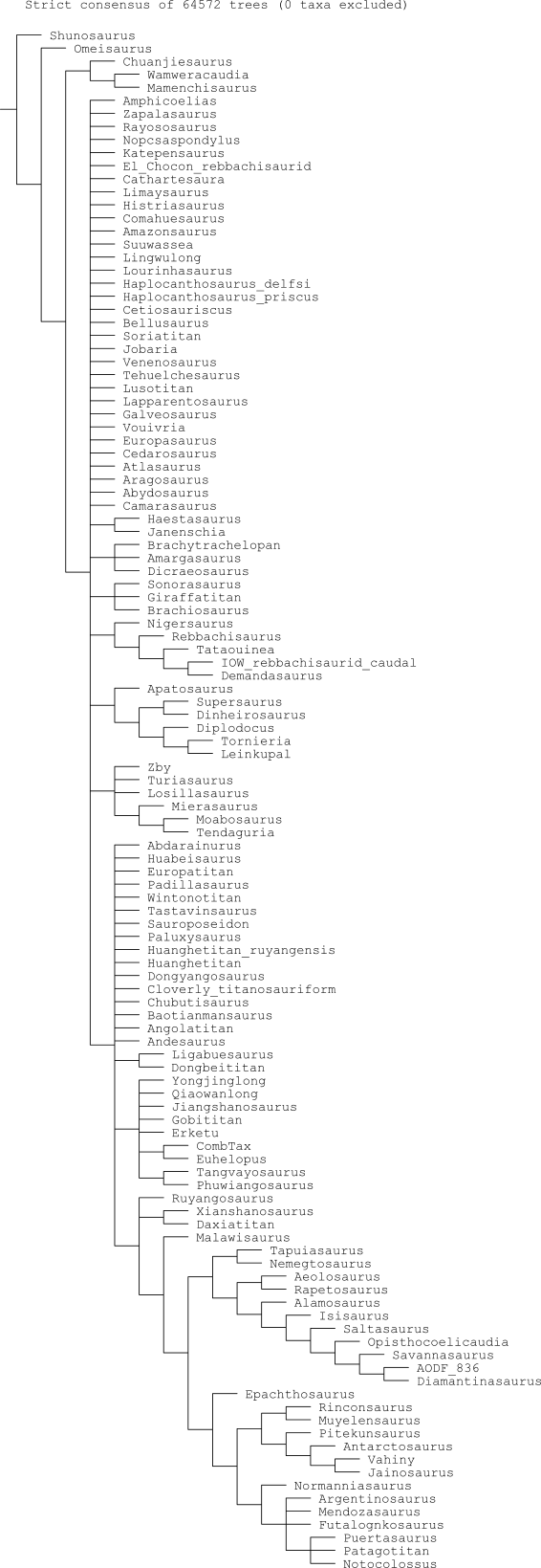

Supplement: Supplementary file 2 — Supplementary Information 2. [file 41598_2021_94273_MOESM2_ESM.docx]
